# High-content imaging assay to evaluate *Toxoplasma gondii* infection and proliferation: A multiparametric assay to screen new compounds

**DOI:** 10.1371/journal.pone.0201678

**Published:** 2018-08-29

**Authors:** Bastien Touquet, Léonie Pelissier, Pierre Cavailles, Wei Yi, Valeria Bellini, Corinne Mercier, Marie-France Cesbron-Delauw, Ahcène Boumendjel, Delphine Aldebert

**Affiliations:** 1 Institute for Advanced Biosciences, Team Membrane Dynamics of Parasite-Host Cell Interactions, CNRS UMR 5309, INSERM U1209, Université Grenoble Alpes, Grenoble, France; 2 Unité Techniques de l'Ingénierie Médicale et de la Complexité - Informatique, Mathématiques et Applications, Equipe TheREx, CNRS UMR 5525, Université Grenoble Alpes, Grenoble, France; 3 Département de Pharmacochimie Moléculaire, CNRS UMR 5063, Université Grenoble Alpes, Grenoble, France; 4 Unité Techniques de l'Ingénierie Médicale et de la Complexité - Informatique, Mathématiques et Applications, Equipe BNI, CNRS UMR 5525, Université Grenoble Alpes, Grenoble, France; 5 Institute for Advanced Biosciences, Team Host-Pathogen Interactions and Immunity to Infection, CNRS UMR 5309, INSERM U1209, Université Grenoble Alpes, Grenoble, France; 6 Unité Techniques de l'Ingénierie Médicale et de la Complexité - Informatique, Mathématiques et Applications, Equipe GEM, CNRS UMR 5525, Université Grenoble Alpes, Grenoble, France; University of Georgia, UNITED STATES

## Abstract

*Toxoplasma gondii* is an intracellular protozoan parasite widely distributed in animals and humans. Infection of host cells and parasite proliferation are essential steps in *Toxoplasma* pathology. The objective of this study was to develop and validate a novel automatic High Content Imaging (HCI) assay to study *T*. *gondii* infection and proliferation. We tested various fluorescent markers and strategies of image analysis to obtain an automated method providing results comparable to those from gold standard infection and proliferation assays. No significant difference was observed between the results obtained from the HCI assay and the standard assays (manual fluorescence microscopy and incorporation of [^3^H]-uracil). We developed here a robust and time-saving assay. This automated technology was then used to screen a library of compounds belonging to four classes of either natural compounds or synthetic derivatives. Inhibition of parasite proliferation and host cell toxicity were measured in the same assay and led to the identification of one hit, a thiosemicarbazone that allows important inhibition of *Toxoplasma* proliferation while being relatively safe for the host cells.

## Introduction

*Toxoplasma gondii* is an obligate intracellular protozoan parasite responsible for toxoplasmosis, an infection which is asymptomatic in more than 80% of immune-competent subjects [1]. Severe infections are mainly observed in pregnant women and immune-compromised patients. Severe cases of toxoplasmosis resulting from infection with atypical genotypes of *Toxoplasma* strains have also been recently observed in immune-competent subjects [2].

Despite its clinical importance, only few therapeutic drugs are available to treat infections by *T*. *gondii*. The most common therapeutic medicines used against the parasite are 1) spiramycin in pregnant women and 2) combination therapies such as pyrimethamine-sulfadoxine, pyrimethamine-sulfadiazine and trimethoprim-sulfamethoxazole in immune-deficient patients or in severe cases of toxoplasmosis. However, these treatment regimens cannot always be used due to diverse reasons, such as intolerance, problems related to drug absorption or resistance of the parasite. Moreover, drug toxicity due to sulfadiazine hypersensitivity further complicates the life-long prophylactic treatment of immunocompromised patients as well as treatment during pregnancy [3].

The parasite virulence depends on the capacity of *Toxoplasma* to invade and replicate within its host cells [4] Infection has been analyzed using various methods including β-galactosidase activity assays, microscopic analysis (phase and fluorescence), flow cytometry and quantitative PCR. Proliferation has been analyzed by plaque assays, incorporation of [^3^H]-uracil, quantitative PCR. Some of these assays present the disadvantages of being time-consuming and costly, especially when large series of samples have to be screened [5].

The aim of this study was to develop a High Content Imaging (HCI) assay that allows the analysis of *Toxoplasma* infection and proliferation in one single assay. First, we compared various labeling markers and the novel method with standard procedures including microscopic observation and incorporation of [^3^H]-uracil. Our method was then validated with different parasite strains described as presenting a failure in the infection or proliferation process. Finally, in the context of the discovery of novel and effective molecules against *T*. *gondii* infections, we screened in-house chemical libraries belonging to four chemical categories: two classes of natural products derivatives belonging to polyphenols [6], namely chalcones [7,8] and aurones [9], and two classes of fully synthetic compounds, namely thiosemicarbazones and diphenyloxadiazoles [10]. Our interest towards these four classes of substances was motivated by their drug-like chemical structures [7], their therapeutic potential within the infectious diseases areas and because, to the best of our knowledge, most of them (chalcones, aurones, and diphenyloxadiazoles) had never been the subject of investigations as anti-*Toxoplasma* agents. The targeted compounds were screened for their capacity to inhibit parasite proliferation as well as for their lack of toxicity on host cells in the same assay.

## Material and methods

### Cell cultures and parasites

Human foreskin fibroblasts (HFF) were obtained from the American Type Culture Collection (ATCC, Manassas, VA, USA). Tachyzoites of both the type II Prugniaud- and the type I RH-YFP2 (kindly provided by B. Striepen, Athen, GA, USA) strains were maintained in HFF monolayers in D10 medium (DMEM supplemented with 10% heat-inactivated fetal bovine serum, 1 mM glutamine, 500 units/mL penicillin and 50 μg/mL streptomycin) in a humidified incubator, at 37°C, and under 5% CO_2_. The parasites were collected just after host cell lysis, centrifuged at 800 g for 5 min, suspended in D10 medium, and counted.

### Compounds

A pyrimethamine (Sigma-Aldrich) stock solution was prepared at 10 mM in DMSO and was used at the final concentration of at 20 μM as a positive control for anti-*Toxoplasma* activity. Forty-six molecules were tested to determine their anti-*Toxoplasma* activity. Each molecule was prepared as a 10 mM stock solution in DMSO and tested at 10 μM. The hits were then further tested at the dilutions 0.01 to 100 μM. Hoechst-33342, trihydrochloride, trihydrate (Sigma-Aldrich) was used as a marker of nucleic acids to detect parasites and cell host nuclei. Monoclonal antibody TG17.43 anti-GRA1 (Biotem) and goat anti–mouse IgG (H+L) coupled to Alexa Fluor-488 (Thermofisher) were used to detect *Toxoplasma* parasites and their parasitophorous vacuole. [^3^H]-uracil was used to analyze parasite proliferation.

### Incorporation of [^3^H]-uracil

The intracellular growth of RH-YFP2 parasites in HFF was monitored by selective incorporation of [^3^H]-uracil, as previously described [11]. Briefly, confluent HFF in 24-well plates were infected with 1.6x10^5^ parasites for 2 h in D10 medium, at 37°C, and 5% CO_2_. After several washes to eliminate extracellular parasites, infected cells were cultured for 30 h with 185 Bq of [^3^H]-uracil per well. Monolayers were washed 3 times in phosphate buffered saline (PBS), disrupted with 500 μL of lysis/scintillation solution (Optiphase Supermix, Perkin Elmer, France) and their radioactivity was measured by liquid scintillation counting using a Wallac MicroBeta TriLux (Perkin Elmer) calibrated for [^3^H]-uracil. Two experimental replicates were performed in each of the 3 separate experiments.

### Microscopy assays

#### Host cell infection, drug incubation and staining

Cells were seeded at a density of 1.10^4^ cells per well into 96-well plates, and allowed to grow for 48 h at 37°C and 5% CO_2_ to obtain sub-confluent HFF monolayers. Cells were then infected with 4.10^4^ parasites per well. The plates were centrifuged for 1 min at 200 g and placed at 37°C, 5% CO_2_, for 2 h. After 3 washes in PBS, infected cells were incubated at 37°C, 5% CO_2_, for 30 hours with 200 μL of DMEM complete medium alone or supplemented with anti-*Toxoplasma* molecules, i.e. the reference drug pyrimethamine, or the tested molecules. These tested drugs were incubated at 10 μM in screening hit assays. Then, each active molecule was tested at concentrations ranging from 0.037 to 81 μM in 0.8% DMSO to determine the 50% inhibition concentration (IC_50_). Negative (0.8% DMSO) and positive (pyrimethamine at 20 μM/IC_100_) controls were introduced in each well plate.

Five μg/mL Hoechst-33342 was incubated for 20 min on host cells infected by RH-YFP2-parasites. After 2 washes, the cells were fixed with 3.7% formaldehyde for 10 min at 37°C. In experiments using non-fluorescent parasites, the *Toxoplasma* vacuoles were fixed for 15 min with 3.7% formaldehyde and stained with anti-GRA1 mAb (1/500 in 2% BSA and 0.1% triton-X 100 in PBS) for 1 h at room temperature. After 2 washes, goat serum anti-mouse IgG (H+L)-Alexa-488 (1/500) was added for 20 min. Finally, Hoechst-33342 was added as described above.

#### High content imaging acquisition

Imaging of 96-well plates was performed using a high content imaging system (Olympus ScanR). The 20 X objective (N.A. 0.45) was used to collect images for the distinct fluorescence channels: Hoechst-33342 (ex. 360–370 nm, em. 420–460 nm), YFP2 and Alexa-488 (ex. 460–495 nm, em. 510–550 nm). The exposure times were 20 ms for the Hoechst-33342 channel and 100 ms for both the Alexa-488 and YFP2 channels. Twenty fields per well were imaged.

#### High content image analysis

The acquired images were analyzed using the ScanR analysis software. This analysis aimed to identify the parasitophorous vacuoles (V), the parasites’ nuclei (P) and host cells’ nuclei (C).

**Fig 1:**
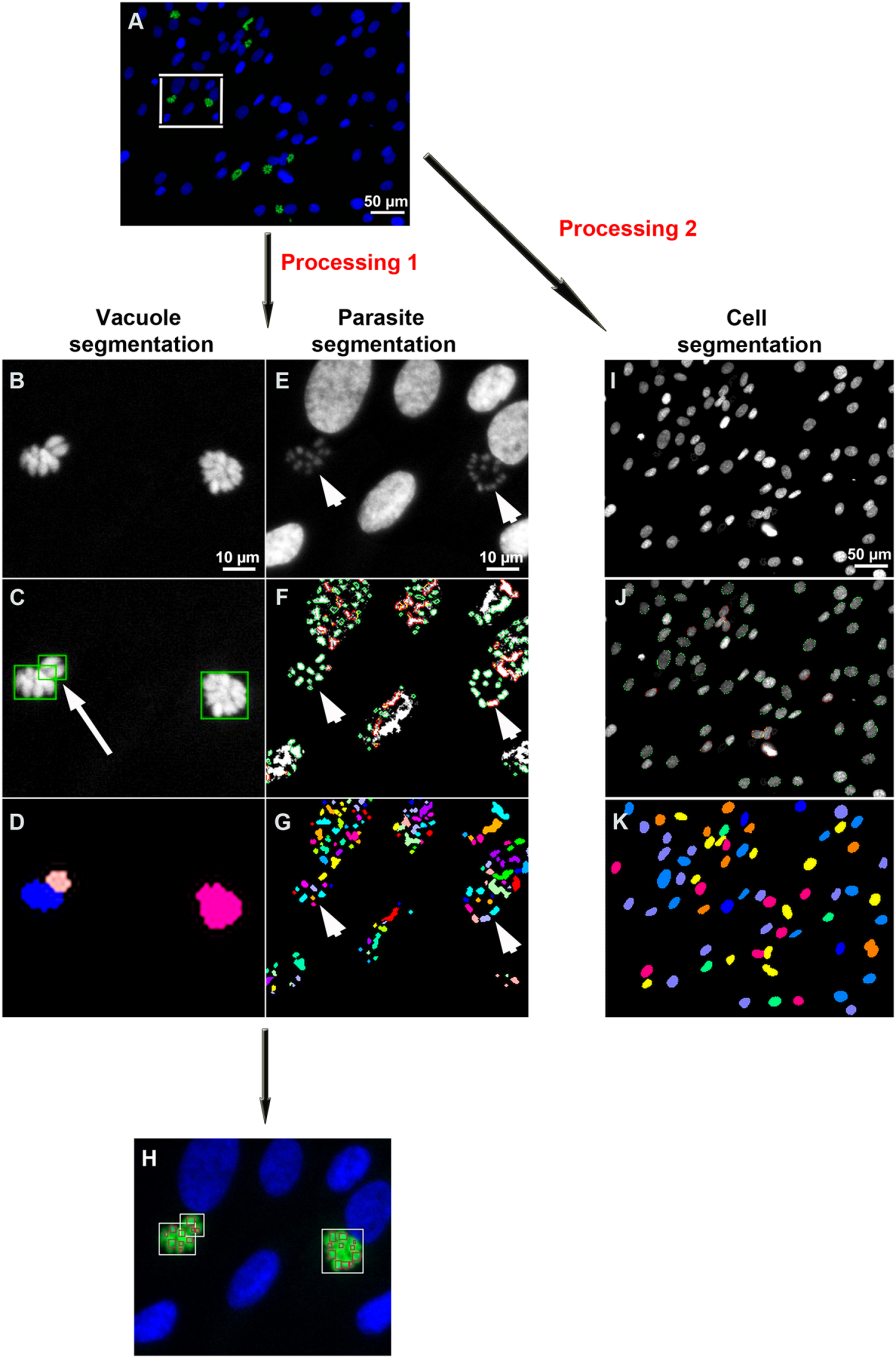
Images processing steps. A: Example of raw image acquired with the automated ScanR Olympus software. B-H: Cropped images to explain the image process analysis. B-D: Image processing to detect *Toxoplasma* parasitophorous vacuoles using the YFP2 /Alexa-488 channel. E-G: Image processing to detect parasites using the Hoechst-33342 channel. C, F: Images were filtered, the background was subtracted and a watershed algorithm was applied to identify both the vacuoles and the parasites. D, G: A binary vacuole mask and a parasite mask were generated. H: Masks were merged to identify parasites inside their vacuoles. I-K: Host cell nuclei processing. I: Host cells were detected using the Hoechst-33342 channel. J: Images were filtered, the background was subtracted and an edge algorithm was used to segment host cells. K: A binary host cell mask was generated.

*Segmentation of the parasitophorous vacuoles and the parasites’ nuclei*: *Processing 1*: Raw images (Fig 1A) were acquired with the ScanR analysis software developed by Olympus (Germany, Heildelberg, EMBL). In the first image processing (Fig 1B–1H), segmentation of the parasitophorous vacuoles and that of the parasites (Fig 1B–1H) were processed independently from the same raw images and merged to identify individual vacuoles and their assigned parasites. The segmentation of the parasitophorous vacuoles (Fig 1B–1D) was performed from the pixels of parasite YFP fluorescence (Fig 1B) or Alexa 488-immunolabeled GRA1 (not shown). A fixed threshold was set up (Fig 1C) and a watershed algorithm was applied to split apart two vacuoles that associated with one another (Fig 1C, white arrow). A binary mask was obtained (Fig 1D) and objects on the image border were excluded. Segmentation of the parasites’ nuclei (Fig 1E–1G, arrowhead) was performed with the Hoechst fluorescence pixels (Fig 1E). In order to properly separate parasites’ nuclei one from another, an important rolling ball background subtraction filter was applied (5 pixels). Both the brightness and the contrast of filtered images were adjusted manually. Parasites were selected with an edge segmentation algorithm with a minimum object size of 10 pixels and a maximum object size of 30 pixels (Fig 1F). The obtained binary image allowed a clear detection of the parasites (Fig 1G).

The vacuole mask was merged with that of the parasites to identify parasites inside their parasitophorous vacuole and to exclude extracellular parasites or host cell nuclei (Fig 1H).

*Segmentation of the host cells’ nuclei*: *Processing 2*: The second processing aimed to identify the host cell nuclei (Fig 1I–1K). Host cell segmentation consisted in adjusting the brightness and the contrast of all set images (Fig 1I) before using an edge algorithm to detect the periphery of each host cell nucleus (Fig 1J). A watershed algorithm was applied on the obtained binary image to split cells apart from each other (Fig 1K).

#### Manual microscopy analysis

Parasitophorous vacuoles, parasites and host cells were manually counted on the set of images provided by the Olympus IX81 microscope. Three different experiments were performed.

### Data analyses

Several data per well were extracted and used to determine:

1. the *T*. *gondii* infection rate (V/C), calculated as the ratio between the number of parasitophorous vacuoles per well (V) divided by the number of host cells per well (C);
2. the *T*. *gondii* proliferation index (P/V), calculated as the ratio between the number of parasites per well (P) divided by the number of parasitophorous vacuoles per well;
3. the intra-vacuolar distribution of parasites (percentage of parasitophorous vacuoles containing either 1, 2, 4, or 8 parasites);
4. the parasite proliferation inhibition (PPI), calculated with the following formula:
  PPI=(1−((P/V[mol]−P/V[Pyr])/(P/V[DMSO]−P/V[Pyr])))x100
  where P/V_[Pyr]_ was the average proliferation rate of *Toxoplasma* in the presence of 20 μM pyrimethamine, P/V_[DMSO]_, the average proliferation rate of *Toxoplasma* in the presence of DMSO only and P/V_[mol]_, the average proliferation rate of *Toxoplasma* in the presence of the tested molecule;
5. the cell viability (CV): (C _[mol]_ / C_[DMSO]_)x100, where C_[mol]_ was the number of host cell nuclei per well containing the tested molecule and C_[DMSO]_, the number of host cell nuclei per well containing the DMSO control.

Both the 50% of parasite proliferation inhibition (IC_50_) and the 50% of cell viability (CV_50_) of each tested molecule were determined by fitting their dose-response curve using the GraphPad Prism software. A selectivity index activity (SI) was calculated as SI = CV_50_/IC_50_.

### Assay quality control

Validation of the HCI assay was assessed with repeatability and reproducibility tests, and calculation of the Z’ factor [12]. Briefly, the condition corresponding to the maximal fluorescent signal was used as positive control while that corresponding to the minimal fluorescent signal was used as negative control. Each Z’ factor plate contained 48 replicate negative controls and 48 replicate positive controls. Both the mean and the standard deviation were used to determine the Z’ factor as = [3 SD of positive control + 3 SD of negative controls] / [mean of positive controls–mean of negative controls]. The pipetting variability and edge effects were assessed by the evaluation of the cell number in three different plates in which the cells had been treated with 0.8% DMSO or 20 μM Pyrimethamine. Inhibition of parasite proliferation was analyzed in three 96 well-plates in presence of 20 μM pyrimethamine or 0.8% DMSO to calculate the Z’ factor. The IC_50_ of the 20 μM concentration in pyrimethamine was determined at 3 different days to assess the IC_50_ reproducibility.

### Statistical analyses

The data generated by gold standard methods and compared to those obtained from the HCI assay were expressed as means ± SD and the significance of differences found between groups was determined using the two-tailed Student’s *t* test (Prism software).

## Results

### Validation of High Content Imaging assay in comparison with gold standard assays

Human foreskin fibroblasts are currently used as a host model to study *Toxoplasma* infection *in vitro*. We optimized our cell culture and parasitophorous vacuoles’ labeling conditions to obtain efficient segmentation of both the vacuoles and the parasites in order to develop a High-Content Imaging assay (HCI) for the evaluation of *Toxoplasma* infection and proliferation. We optimized the HFF cell seeding density (1.10^4^ per well) in 96 well plates to obtain a sub-confluent monolayer after 48 h of culture, which allowed satisfying segmentation of the host cells: the number of cells containing at least one *Toxoplasma* parasitophorous vacuole was > 15% following the inoculation of 4.10^4^ parasites let to invade for 2 hours.

Both the host cells and the parasites were identified and counted using the nucleic acid marker Hoechst-33342. However, when directly counted, it was sometimes difficult to discriminate parasites’ nuclei from host cells’ nuclei. Counting the parasites in each cell was facilitated by a first segmentation of the vacuoles.

The segmentation process used to determine the infection rate and assess *Toxoplasma* proliferation was performed as described in the material and methods (Fig 1).

**Fig 2:**
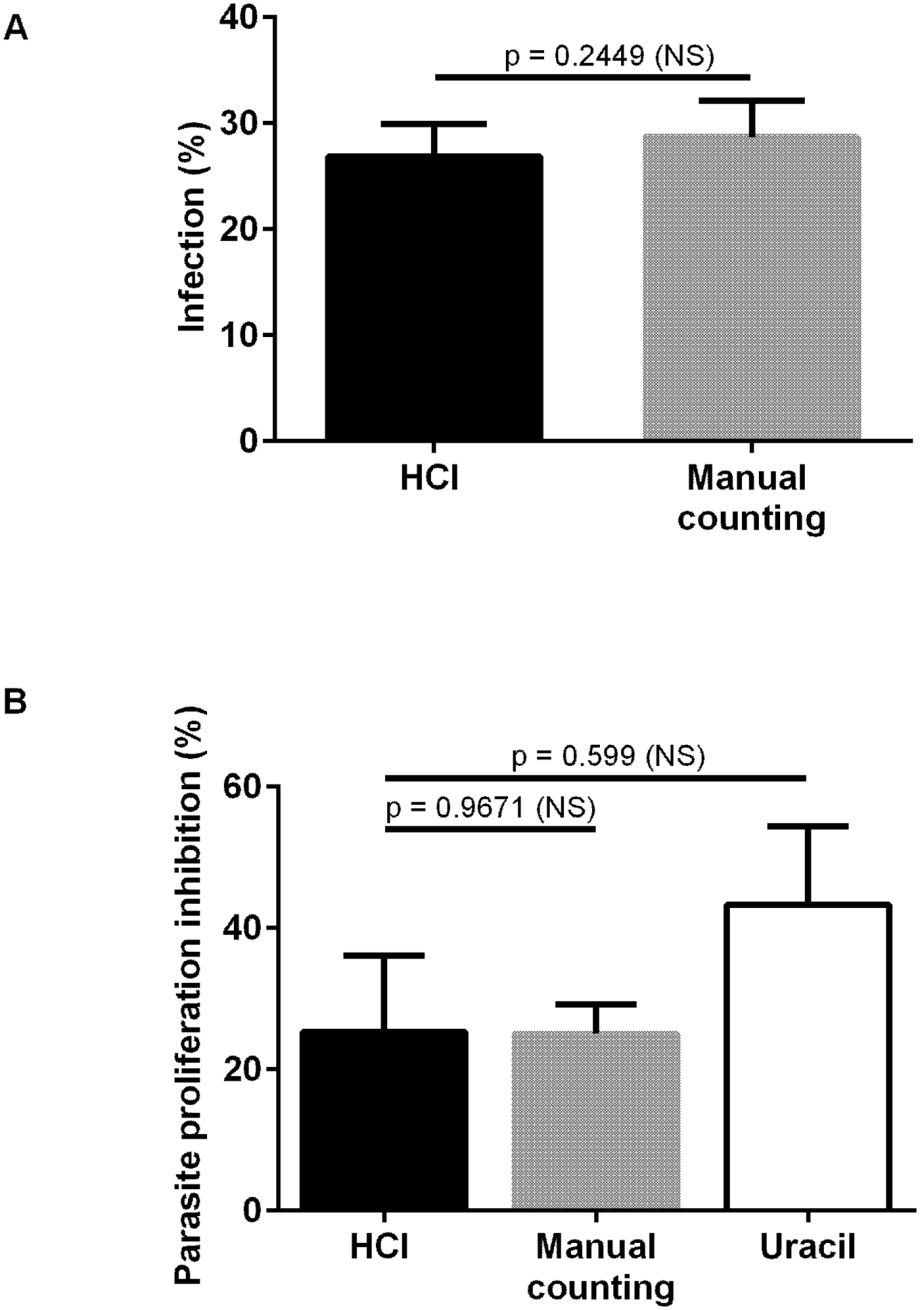
Comparison between the infection- and proliferation rates as determined by the HCI- *versus* the gold standard assays. Vacuoles and parasites were counted to determine both the infection- and the parasite proliferation rates. Histograms represent the mean +/- standard deviation of 3 independent experiments. A-Comparison of *Toxoplasma* infection rates, as determined by the HCI assay *versus* manual counting. B- Comparison of *Toxoplasma* proliferation, as determined by the HCI assay *versus* standard methods, *i*.*e*. manual microscopic counting and the [^3^H]-uracil incorporation assay, respectively.

We then compared the infection rates as determined from the HCI assay *versus* manual microscopic observations. No difference was observed (Fig 2A). Three experiments were performed and the infection rates were respectively 26.66 ± 3.06 (HCI) and 28.72% ± 3.42 (manual counting), leading to a high correlation (r = 0.99) (Fig 2A).

After 30 h of parasite culture in HFF, the total numbers of parasitophorous vacuoles and parasites were counted in wells with or without 20 μM pyrimethamine from 3 different experiments. The 100% proliferation corresponds to the number of parasites per well without pyrimethamine. The inhibition of parasite proliferation, as determined by HCI, manual microscopic observations and [^3^H]-uracil incorporation assay, was respectively 25.2% ± 10.8, 25% ± 4.2 and 43.25% ± 11.2. There was thus no significant difference between the results from HCI *versus* manual counting after microscopic observation of 20 fields (p = 0.9671) or [^3^H]-uracil incorporation assay (p = 0.599) (Fig 2B).

### Validation of the HCI assay using standard *T*. *gondii* strains

**Fig 3:**
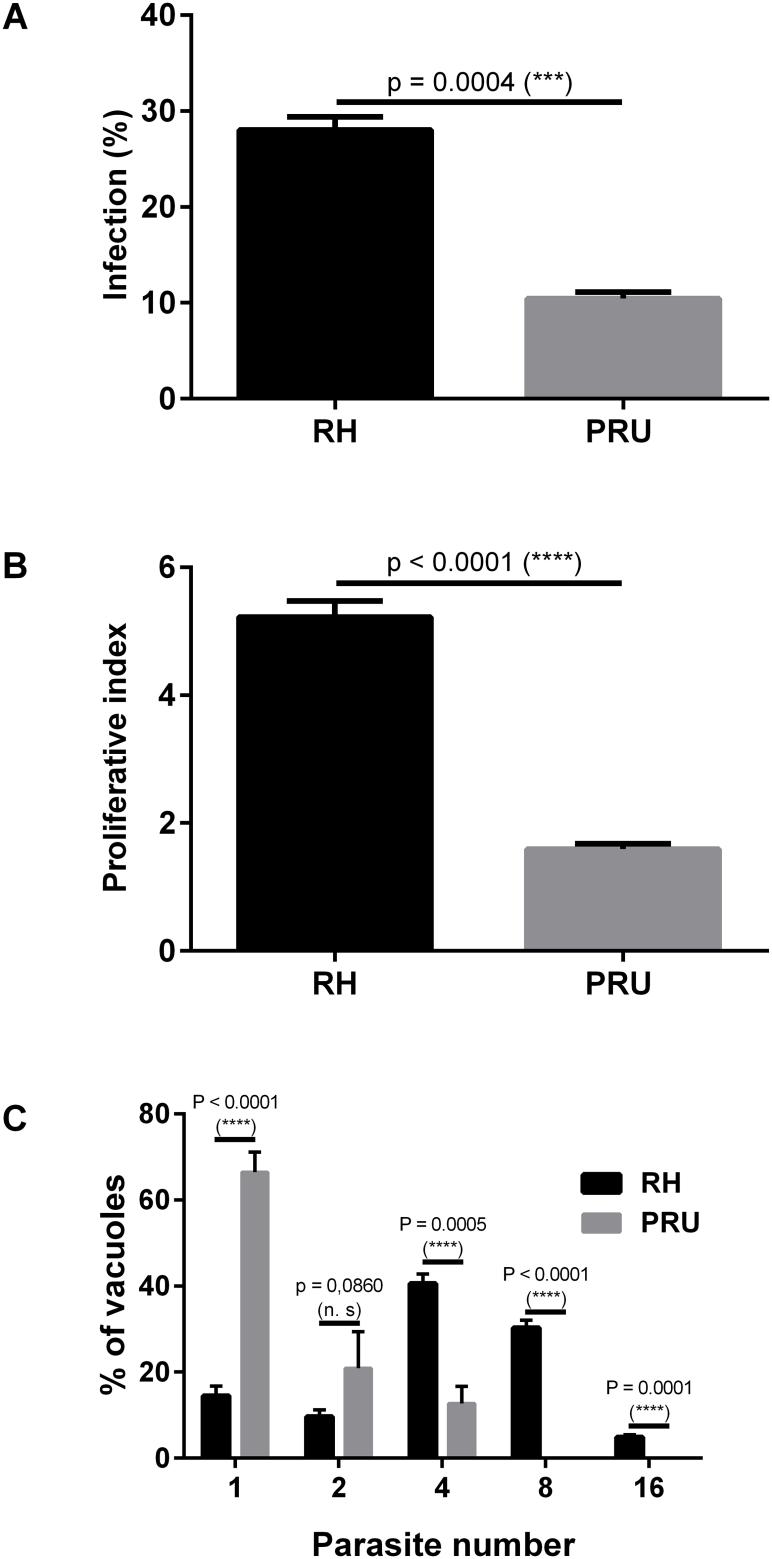
Comparison of the infection- and the proliferation rates of RH-YFP2 *versus* PRU *Toxoplasma* strains. Vacuoles and parasites were counted to determine both the infection- and the proliferation indexes. Histograms represent the mean +/- standard deviation from 3 independent experiments. A-Comparison of the infection rates of RH-YFP2 *versus* PRU B- Comparison of the proliferation indexes of RH-YFP2 *versus* PRU C- Comparison of the number of RH-YFP2 *versus* PRU parasites (1, 2, 4, 8, or 16) per vacuole after 30 h of development within HFFs.

We then analyzed with our HCI assay the phenotype of 2 *Toxoplasma* strains (RH and PRU) described in the literature to have different infection- and proliferation rates. The infection rates of RH *versus* PRU were respectively 28.3% *versus* 10.5% (Fig 3A). The proliferation indexes were respectively 5.2 ± 0.24 (RH) *versus* 1.6 ± 0.08 (PRU) (Fig 3B). Unlike [^3^H]-uracil incorporation assay, the HCI assay allowed the analysis of the proliferation in each vacuole. After 30 h of incubation, we observed that more than 80% of the vacuoles infected by RH contained more than 2 parasites, whereas less than 40% of the PRU vacuoles contained more than 2 parasites (Fig 3C).

### Repeatability, reproducibility and robustness of the HCI assay

**Fig 4:**
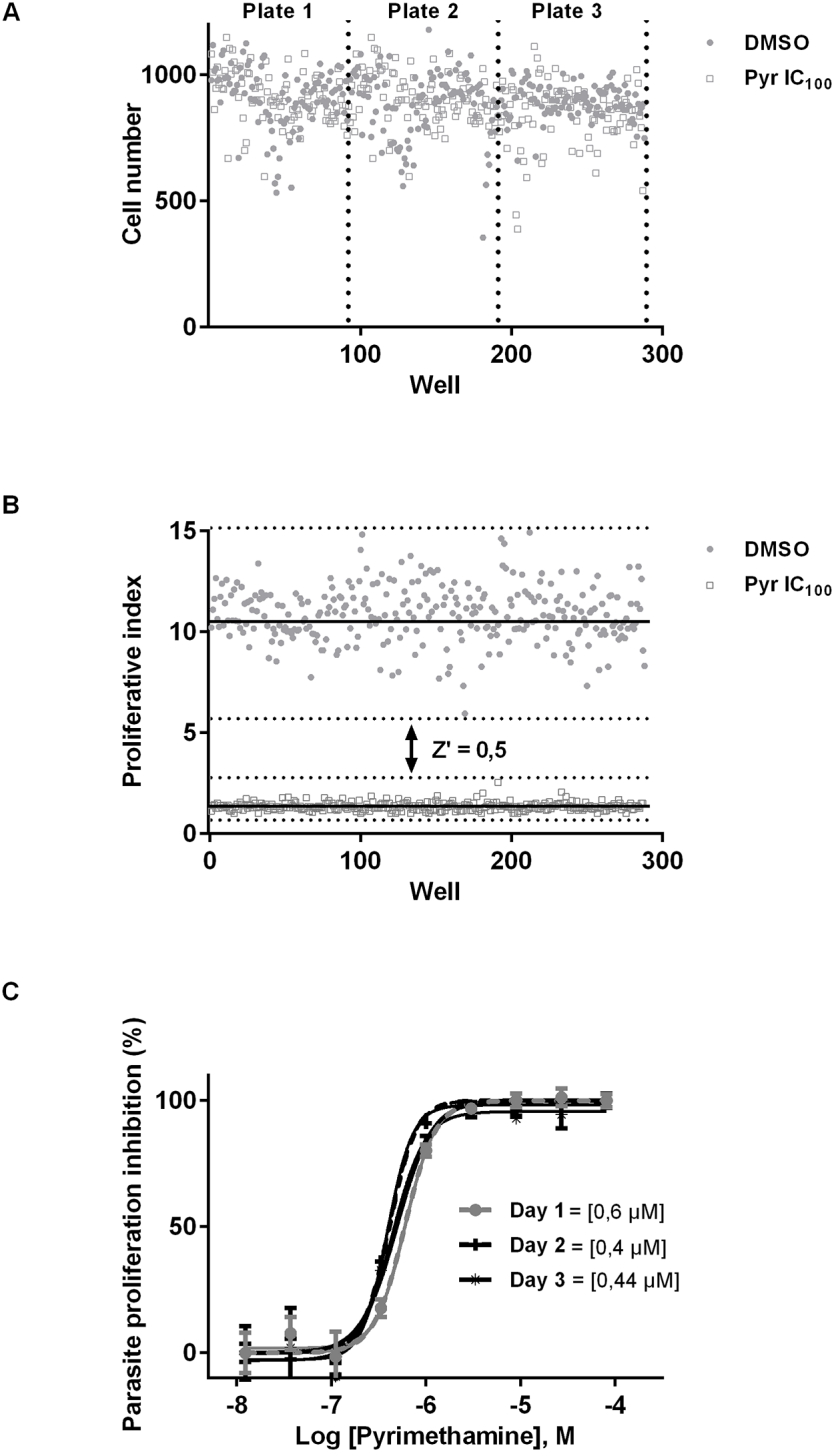
Repeatability, reproducibility and robustness of the HCI assay. A-Plot of the number of host cells in each well of a set of 3 96-well plates to calculate the intra-plate and inter-plate variation. HFFs incubated with DMSO were represented with black dots while those incubated with pyrimethamine were represented with white squares. B- Plot of *Toxoplasma* proliferation to assess the robustness. HFF incubated with pyrimethamine (white squares) or DMSO (black dots). The Z’ factor was also calculated. C-Pyrimethamine dose-response curve and determination of the IC_50_ at 3 different days to calculate reproductibility. HFFs were incubated with *Toxoplasma* parasites and pyrimethamine. Vacuoles and parasites were counted to calculate the parasite proliferation inhibition and to determine the IC_50_.

Repeatability and reproducibility of the HCI were determined by measuring the coefficient of variation of cell number after *Toxoplasma* infection and treatment with DMSO or pyrimethamine. First we analyzed the number of HFF on 20 image fields per well within a 96-well plate and across multiple plates. The intra-plate variation coefficients for DMSO and pyrimethamine-treated wells were respectively 12.1% and 13.5%, while the inter-plate variation coefficients were 1.9% and 4.7%, respectively. These results showed an acceptable intra- and inter-plate repeatability of cell numbers (Fig 4A).

The robustness of this assay was assessed by calculating the Z’ factor, as described by Boveia *et al*., 2009. Six plates were scanned and the proliferation index was calculated to determine the Z’ factor based on comparison of the parasite numbers between wells treated with or without pyrimethamine. The obtained z’ factor (≥ 0.5) demonstrated the robustness of the HCI assay (Fig 4B).

One of the objectives of this study was to apply the HCI assay to the screening of potential new therapeutics against *Toxoplasma*. We thus validated the reproducibility of our HCI assay at 3 different days by calculating the IC_50_ of pyrimethamine. The IC_50_ were respectively 0.60; 0.40 and 0.44 μM confirming the reproducibility and robustness of the HCI assay (Fig 4C).

### Screening of potential anti-*Toxoplasma* compounds

**Fig 5:**
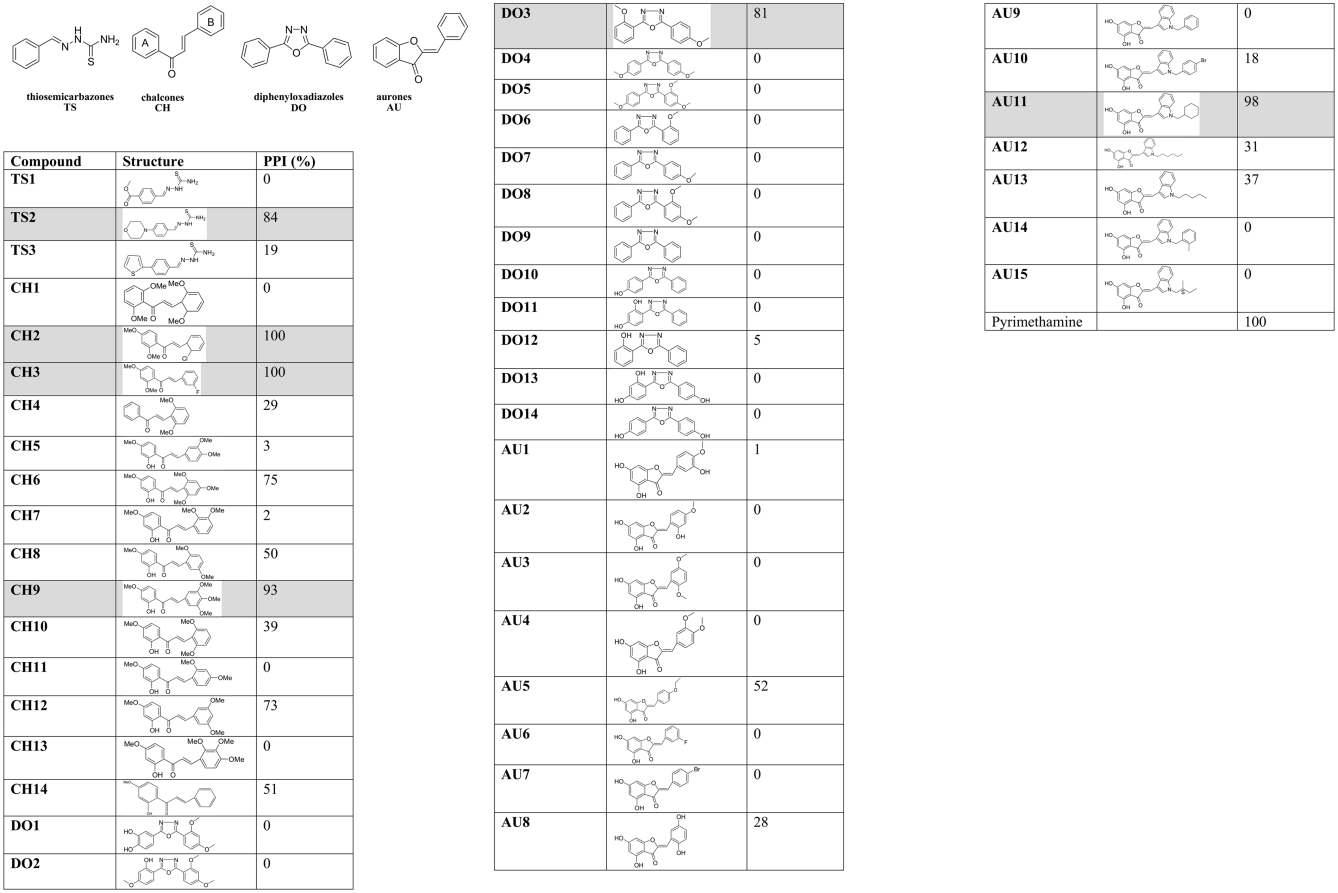
Inhibition of *T*. *gondii* proliferation by 46 molecules. Parasite proliferation inhibition (PPI). The rows shaded in grey highlight the hit molecules.

The screening of molecules never tested on *T*. *gondii* aims to identify new compounds able to inhibit *in vitro* proliferation of *T*. *gondii* in HFF without toxicity against the host cells. Therefore, both inhibition of parasite proliferation and host cell viability were assessed using the HCI assay. The percentage of parasite proliferation inhibition was reported in Fig 5.

Six compounds, *i*.*e*. 1-thiosemicarbazone (**TS2**), 3-chalcones (**CH2**, **CH3** and **CH9**), 1-dipehyloxadiazol (**DO3**) and 1-aurone (**AU11**), respectively, led to a high proliferation inhibition activity. Following the primary screening, these 6 compounds were tested for dose-response studies as well as cell viability assays (Table 1). Their IC_50_ was comprised between 0.9 and 27.7 μM, leading to selectivity indexes comprised between 21 and > 90 (Table 1). The most active and less toxic hit, *i*.*e*. the thiosemicarbazone (**TS2**), showed characteristics (IC_50_: 0.9 μM; CV_50_ > 81 μM; SI >90) comparable to those of the pyrimethamine reference drug (IC_50_: 0.4 μM; CV_50_ > 81 μM; SI >202).

**Table 1:** *T*. *gondii* proliferation inhibition activity and cytotoxicity of the six identified hits.

Compound	IC_50_ (μM)^a^	CV_50_ (μM)^b^	SI^c^
**TS2**	0.9 ± 0.1	>81	>90
**CH2**	3.3 ± 0.2	31.5 ± 7.3	9.5
**CH3**	3.7 ± 0.2	7.8 ± 2.5	2.1
**CH9**	6.6 ± 1.5	>81	>12.3
**DO3**	27.7 ± 6.2	>81	>3
**AU11**	9.9 ± 2.4	>81	>8.2
**pyrimethamine**	0.4 ± 0.03	>81	>202

^a^IC_50_: concentration (μM) to inhibit 50% of *T*. *gondii* proliferation

^b^CV_50_: concentration (μM) to inhibit the viability of 50% of host cells

^c^selectivity index: CV_50_/IC_50_

## Discussion

High-content imaging is a rapidly growing domain that has shown great potential, especially in the microbiology field. In the present study, we developed a high-content imaging assay for parasitology and especially for the detection and quantification of intracellular *Toxoplasma* parasites. This technology has already been developed for other protozoan parasites such as *Plasmodium*, *Leishmania and Trypanosoma* [13–17]. In the latter context, most efforts were focused on software development. In a distinct way, our objective was not to develop an algorithm for parasite detection. Our goal was instead to work out the cell culture, parasite infection and staining protocols to optimize the analysis process.

Our first goal was to count the number of parasites accurately. Reporter genes have been successfully developed for HCI by various groups, in particular for drug screening [13]. However, a DNA marker is necessary to detect the wild-type parasites and some authors have reported that engineered parasites could exhibit modified infection- and/or proliferation rates, or loss of transgene expression [16]. On the opposite, Siqueira-Neto *et al*. [16] reported the effectiveness of DNA labeling in high content imaging to detect and quantify *Leishmania*. Hence, we tested both YFP2- and Hoechst-33342-labelled parasites. The latter were segmented in a more efficient manner, allowing a first segmentation on parasitophorous vacuoles. This first step of segmentation proved to be particularly useful when parasites were close to the host cell nucleus, such as in cells with limited cytosol (THP-1, macrophages) or when multi-infection was observed. The use of a parasitophorous vacuole marker such as GRA1 [18] allowed to improve the quality of the results.

Even if HCI has already been used to understand the process of *Toxoplasma* infection [19], this method had never been compared to the standard *Toxoplasma* assays used to quantify parasite infection and proliferation. Here, we demonstrated that this HCI assay provides results comparable to those obtained by manual microscopic counting and radioactive method. HCI is an accurate method compared to manual microscopy since it provides more statistically representative data through the capture of a large number of host cells. The most significant advantage of HCI over manual microscopic counting relies in its highly reduced processing time. HCI is an accurate method compared to the [^3^H]-uracil incorporation assay because HCI provides the measure of several parameters at the same time. Inhibition of parasite proliferation in host cells can be due to the absence of parasite multiplication or to the loss of host cells. To avoid this potential bias, the HCI method takes both these parameters in consideration: it is possible to count the number of intracellular parasites in parallel to the number of host cells, and further proceed with normalization of the number of intracellular parasites to the number of host cells per well. Such normalization cannot be easily performed using standard proliferation assays such as the [^3^H]-uracil incorporation assay.

In this study, we also demonstrated that HCI is an interesting assay to screen potential therapeutic compounds. Even if the time necessary to obtain data is longer than that necessary when using classical assays such as [^3^H]-uracil incorporation or spectrofluorimetry, the HCI assay allows the analysis, in a single test, of both the anti-*Toxoplasma* activity and the host cell cytotoxicity of tested compounds. This latter is particularly important for compounds that may induce necrosis, apoptosis, or which may interfere with the adherence of host cells. We would thus recommend to perform a first screen using [^3^H]-uracil incorporation or spectrofluorimetry to quickly identify the hits, followed by an HCI assay to check the absence of bias and understand the mechanism of action.

Our goal here was to provide hit compounds effective against *T*. *gondii* while having low host cell toxicity. These drugs will be further chemically modified in pursuing the pharmacomodulation and development processes. The compounds tested here were chosen because they offer high chemical diversity, and they possess a high potential of pharmacomodulation. We obtained six interesting hits, namely: 1 thiosemicarbazone (**TS2**), 3 chalcones (**CH2**, **CH3** and **CH9**), 1-dipehyloxadiazol (**DO3**), and 1-aurone (**AU11**). From the observed activities, some structure-activities relationships may already be highlighted in the chalcones series. One structural element that seems determinant for the inhibition activity is the presence of a halogen atom at their B-ring. Interestingly, pyrimethamine, the reference drug, possesses a chlorine atom. It should be noted that the methoxylation pattern of the chalcones is important for inhibition *versus* cytotoxicity. The latter behavior has been observed for chalcones in other pharmacological areas [8,20]. Based on the calculated selectivity index, it can be concluded that the most active and less toxic hit is thiosemicarbazone **TS2**, which exhibits a profile comparable to that of the pyrimethamine reference drug. For further investigation, it will be straightforward to investigate TS2 analogs as well as chalcones derivatives.

The synthesis easiness (1 to 2 steps for TS2 analogs) and the availability of diverse starting blocks required to build both pharmacophores should allow the synthesis of large libraries of analogs, which will ultimately lead to the discovery of more active and less toxic compounds, suited for further development.

## Conclusions

*Toxoplasma gondii* infections constitute a challenging and life-threatening health problem in immunocompromised individuals. The emergence of parasite resistance against the few available molecules and the intolerance to the commonly used anti-*T*.*gondii* drugs during long-term therapy prompt the search for novel anti-*Toxoplasma gondii* agents. In this perspective, the development and the validation of this HCI assay to analyze *Toxoplasma* proliferation is a relevant and effective method to investigate new anti-*Toxoplasma* molecules.
